# A systematic review and meta-analysis of the association between sarcopenia and myocardial infarction

**DOI:** 10.1186/s12877-022-03712-1

**Published:** 2023-01-06

**Authors:** Feika Li, Tingting Bai, Yan Ren, Qiqi Xue, Jiaan Hu, Jiumei Cao

**Affiliations:** Department of Geriatrics, Ruijin Hospital, Shanghai Jiaotong University School of Medicine, Shanghai, China

**Keywords:** Sarcopenia, Myocardial infarction, Systematic review, Meta-analysis

## Abstract

**Background:**

Systematic review and meta-analysis of the association between sarcopenia and the development of myocardial infarction.

**Methods:**

PubMed, Cochrane Library, and Embase were searched for studies on the association between sarcopenia and myocardial infarction from their inception until November 26, 2022. The fixed-effects model was used to calculate the combined risk ratio (RR) of sarcopenia in patients with myocardial infarction. Sensitivity analysis was used to test the robust of the combined result, and funnel plot were used to test publication bias.

**Results:**

Five studies were included finally. There was no significant association between sarcopenia and risk of developing myocardial infarction [RR = 1.01; 95% CI = 0.94, 1.08; *P* = 0.317]. The sensitivity analysis showed robust of the combined result. The funnel plot showed no significant publication bias.

**Conclusion:**

Limited evidence suggests no definitive association between sarcopenia and risk of myocardial infarction.

**Supplementary Information:**

The online version contains supplementary material available at 10.1186/s12877-022-03712-1.

## Highlights

• First meta-analysis on the correlation between sarcopenia and myocardial infarction

• A certain relationship exists between sarcopenia and myocardial infarction

• Provides a clinical basis for sarcopenia patients with myocardial infarction

**Supplementary Information:**

The online version contains supplementary material available at 10.1186/s12877-022-03712-1.

## Background

Sarcopenia is defined as a clinical syndrome with symptoms, such as age-related decrease of skeletal muscle mass, loss of muscular strength, and dysfunction [1]. Sarcopenia has an estimated prevalence rate of 5% to 15%, affecting the population above 65 years [2]. According to epidemiological research, the prevalence of sarcopenia in elderly Asian patients is high variable, ranging from 7.8% to 35.3% [3, 4], displaying a lower rate compared to the Western population [5]. Notably, sarcopenia is strongly associated with impaired physical function [6], poor quality of life (QoL) [7], and higher risk of adverse events (AEs) [8], even death [2]. Studies further showed that sarcopenia increases the likelihood for elderly people to use hospital care, raising the expense of health service [9, 10].

Besides the adverse effects described above, studies reported that sarcopenia is closely related to a higher risk of cardiovascular mortality [11], which is a worldwide disease that causes disability and death. Specifically, studies revealed that patients with myocardial infarction faced a greater probability of developing sarcopenia than those without the condition [12]. Another study also reported that early myocardial contractility impairment raises the risk of mortality in patients with coronary heart diseases by affecting functions related to skeletal muscle strength like grip strength and walking speed [13].

The relationship between sarcopenia and chronic heart failure (CHF), and their common pathophysiological mechanisms and treatments have been recent research focus in studies [14]. Studies found that malnutrition, oxidative stress, systemic inflammatory response, and endocrine imbalance appear to be related with sarcopenia and CHF. However, the underlying mechanisms remains elusive and thus needs to be explored [15, 16]. Sarcopenia and CHF both affect the ubiquitin–proteasome system, myogenic inhibitor signaling, and apoptosis in the muscle [17], which may cause decrease in muscle mass and muscle dysfunction. Moreover, patients sarcopenic CHF patients are at higher risk of suffering from left ventricular ejection fraction, frailty, cachexia, mortality and other aggressive diseases [14].

As mentioned above, although the understanding of the association between sarcopenia and cardiovascular diseases is only in the initial phase, it is reasonably to speculate that there is a close association between them. Notably, an in-depth study on this topic may allow early detection of key risk factors for cardiovascular diseases in older patients with sarcopenia, and could also help effectively organize prevention and treatment strategies associated with particular vulnerabilities. Therefore, by clarifying the association between sarcopenia and myocardial infarction, the current systematic review and meta-analysis attempted to determine whether sarcopenia is associated with a higher risk of occurrence of cardiovascular diseases.

## Methods

### Study design

We conducted and then reported the current systematic review and meta-analysis according to the Preferred Reporting Items for Systematic Reviews and Meta-Analyses (PRISMA) statement [18]. However, we did not publicly register a formal protocol for the current systematic review and meta-analysis.

### Eligibility criteria

Inclusion criteria: (1) clinical studies included sarcopenia patients with myocardial infarction; (2) the results of the literature summary can be expressed in terms of the corresponding statistical indicators; (3) the control group was patients with sarcopenia without myocardial infarction; (4) relationship between sarcopenia and myocardial infarction was used as an outcome indicator; and (5) only studies published in English were considered to meet our inclusion criteria.

Exclusion criteria: (1) animal experiments, review literature, conference reports, case reports, reviews; (2) studies with an unclear diagnosis of sarcopenia or myocardial infarction; (3) the control group combined with other cardiac diseases; (4) studies that could not provide disease correlation.

### Literature search

We performed systematic literature search in PubMed, the Cochrane library, and EMBASE to retrieve potentially eligible studies published before February 7, 2022. In addition, we updated literature search weekly to include the latest studies and the search was last updated on 26 November, 2022. Based on the PICO framework, we construct search strategy by combining ‘myocardial infarction,’ ‘sarcopenia’, and their synonyms using Boolean operators. Details of the search strategy are documented in Supplementary Table 1. Furthermore, we also manually checked the reference lists of included studies to identify studies missed by the electronic search.

### Study selection

We used the EndNote X9 software to manage literature. Duplicate studies were first removed by using this software, and then ineligible studies were excluded based on the title and abstract screening. Finally, we determine eligible studies that met our eligibility criteria based on full-text screening of the remaining studies.

### Data extraction

Duplicate studies were removed. Two researchers (FKL and JMC) independently read the titles and abstracts of the remaining records, excluding those that did not meet the inclusion criteria, and the reasons for exclusion were recorded. Finally, two investigators checked with each other to decide whether to include the reports in the study. Differences in study inclusion were resolved through discussion. If they could not be resolved through discussion, a third researcher (TTB) intervened to decide whether to include the study or not. Data extraction for the included studies was performed independently by two researchers according to a pre-designed table. The extracted contents included: (1) general information: first author, year of publication, nationality, and study type; (2) source of study subjects, diagnostic criteria for sarcopenia and myocardial infarction, myocardial measurements, and infarction grade; (3) outcome indicators: the total number of subjects, the total number of subjects in the “sarcopenia group” and the “non-sarcopenia group”, and the number of subjects complicated by myocardial infarction; (4) the quality evaluation of the literature included in the study was completed by two investigators independently, and the results were checked. If there was any disagreement, it was resolved through consultation and discussion. For studies without data, we contacted the authors by email to obtain the necessary data.

### Quality evaluation

The Quality evaluation for Newcastle–Ottawa Scale (NOS) was used to assess the quality of the included studies [19]. In this tool, seven items were covered, with a total score ranging from 0 to 9. A study was considered to be of high methodological quality if the total score was ≥ 5 stars.

### Statistical analysis

Meta-analysis was performed using STATA 14.0. Statistical heterogeneity among the included studies was evaluated using the Cochrane’s *Q* statistic [20] and *I*^*2*^ index [21]. If *P* > 0.1 and *I*^*2*^ < 50.0%, statistical heterogeneity between studies were considered significant, so the fixed-effects model was used for meta-analysis. On the contrary, statistical heterogeneity was considered insignificant if *P* < 0.1 and *I*^*2*^ ≥ 50.0%, so the random-effects model was used for meta-analysis. The risk ratio (RR) with corresponding 95% confidence interval (CI) was used to express the combined result. When the RR was greater than 1 or the lower limit of the 95% CI was greater than 1 and the diamond box was on the right side of the equivalence line, indicating that the risk of myocardial infarction was higher in the sarcopenic group than in the non-sarcopenic group.

### Sensitivity analysis and publication bias

We performed sensitivity analysis using the leave-one-out strategy to test the robustness of the pooled result. The risk of publication bias was tested by visually inspecting whether the funnel plot was symmetrical, and if both sides of the funnel plot were symmetrical, it indicated that there was no publication bias, while the opposite suggested that there might be some degree of publication bias [22]. When the number of included studies was ≥ 10, Begg’s and Egger’s tests were introduced to quantitatively test whether the funnel plot was symmetrical [23].

## Results

### Literature search

**Fig. 1:**
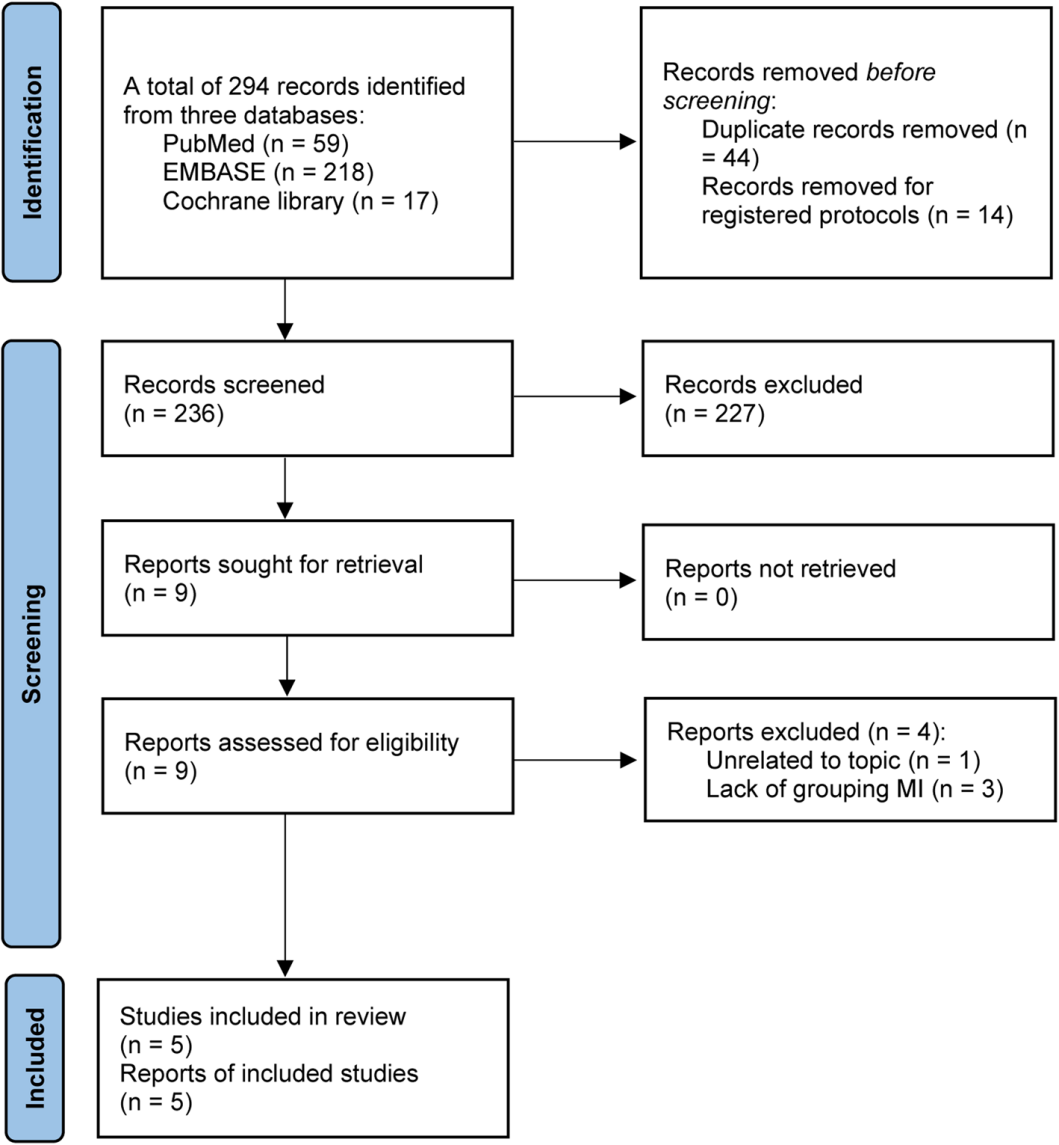
PRISMA flow chart of study screening

The literature search was last updated on 26 November, 2022. A total of 294 studies on sarcopenia and myocardial infarction were retrieved, including 59 studies in PubMed, 17 in the Cochrane library, and 218 studies in Embase. A total of 44 duplicate studies were first removed. Furthermore, 14 registered protocols were also removed. After reading titles and abstracts of the remaining studies, we excluded 227 studies that did not meet our eligibility criteria. After screening full texts of the remaining 9 studies, we further excluded 4 ineligible studies according to the following two reasons: unrelated to the topic (*n* = 1) [24] and lack of grouping myocardial infarction (*n* = 3) [25–27] (Supplementary Table 2). Finally, five studies [28–32] were included in the final data analysis. Following the PRISMA flowchart [18], we showed the study screening process in Fig. 1.

### Basic characteristics of the included studies

The detailed basic characteristics of these five studies are summarized in Table 1. Of these five included studies, two [28, 32] were from China, one[29] was from Brazil, and two [30, 31] were from Japan. Three studies [30–32] used Asian Working Group for Sarcopenia (AWGS) as a diagnostic criterion for sarcopenia [33], which was assessed by dual-energy X-ray absorptiometry and appendicular skeletal muscle mass index (ASMI). However, another two studies [28, 29] made a diagnosis of sarcopenia based on muscle mass, muscle strength and physical performance. For the diagnosis of heart attack, five studies used a comprehensive clinical diagnosis, including serum, electrocardiogram (ECG), and clinical presentation.

**Table 1:** The basic characteristic of the included studies

Author	Country	Age	Study design	Sample	Muscle measurement method	Diagnosis of sarcopenia	Diagnosis criteria for sarcopenia	Diagnostic criteria for heart attack	Heart Attack Classification
Sato 2021 [31]	Japan	72 ± 1/ 65 ± 13	Retrospective case–control study	303	DXA	ASMI	AWGS	Comprehensive clinical diagnosis	Not mentioned
Sato 2020 [30]	Japan	66 ± 13	Observational cohort study	387	DXA	ASMI	AWGS	Comprehensive clinical diagnosis	Not mentioned
Santana 2019 [29]	Brazil	71.6 ± 7.4	Cross-sectional study	99	Muscle mass, HGS measurements made using a digital dynamometer, and the gait speed test	Muscle mass, muscle strength and physical performance	Based on muscle mass, muscle strength and physical performance	Comprehensive clinical diagnosis	Not mentioned
Xia 2021 [32]	China	62.2 ± 8.4	Prospective cohort study	2432	DXA	ASMI	AWGS	Comprehensive clinical diagnosis	Not mentioned
Chang 2022 [28]	China	≥ 20	Cross-sectional study	780,405	Muscle mass, HGS made using a digital dynamometer, the gait speed test	Muscle mass, muscle strength and physical performance	Based on muscle mass, muscle strength and physical performance	Comprehensive clinical diagnosis	Not mentioned

*DXA* Dual energy X-ray absorptiometry, *HGS* Hand grip strength, *ASMI* Appendicular skeletal muscle mass index, *AWGS* Asian working group for sarcopenia

### Quality evaluation

According to the NOS tool, the total NOS score of 3 studies [30–32] was 7 stars and the total NOS score of two studies [28, 29] was 5 stars, indicating that the overall quality of all studies was high in terms of methodological quality. The results of methodological quality assessment based on the NOS tool are shown in Table 2.

**Table 2:** Newcastle Ottawa scale to assess the quality of the included studies

Study	Selection				Comparability	Outcome			NOS score
	Representative of the cases	Selection of controls	Ascertainment of exposure	Outcome of interest	Comparability of the design or analysis	Assessment of outcome	Sufficient follow-up	Adequacy of follow-up	
Sato 2021 [31]	1	1	1	1	2	1	0	0	7
Sato 2020 [30]	1	1	1	1	2	1	0	0	7
Santana 2019 [29]	0	0	1	1	2	1	0	0	5
Xia 2021 [32]	1	1	1	1	2	1	0	0	7
Chang 2022 [28]	0	0	1	1	2	1	0	0	5

### Correlation between sarcopenia and myocardial infarction

**Fig. 2:**
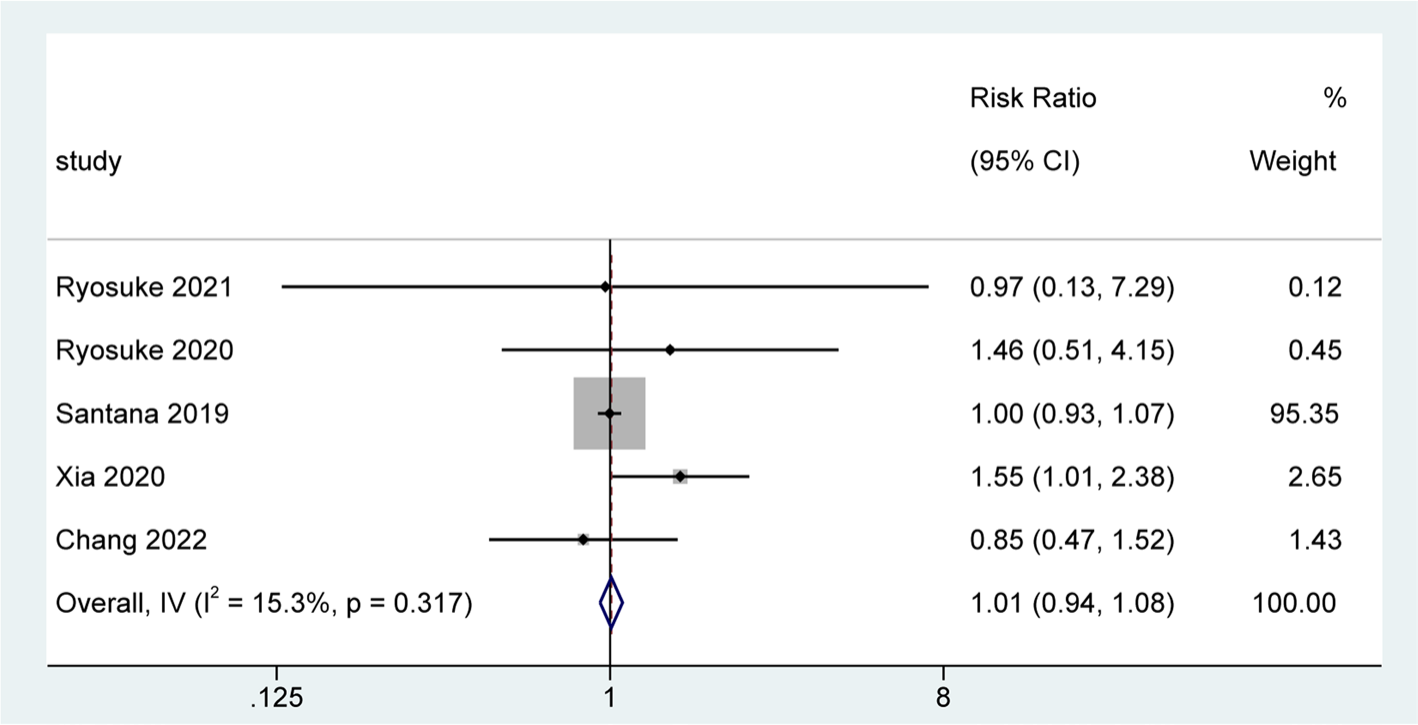
Forest plot of the association between sarcopenia and myocardial infarction, showing a significant association between sarcopenia and risk of myocardial infarction

**Fig. 3:**
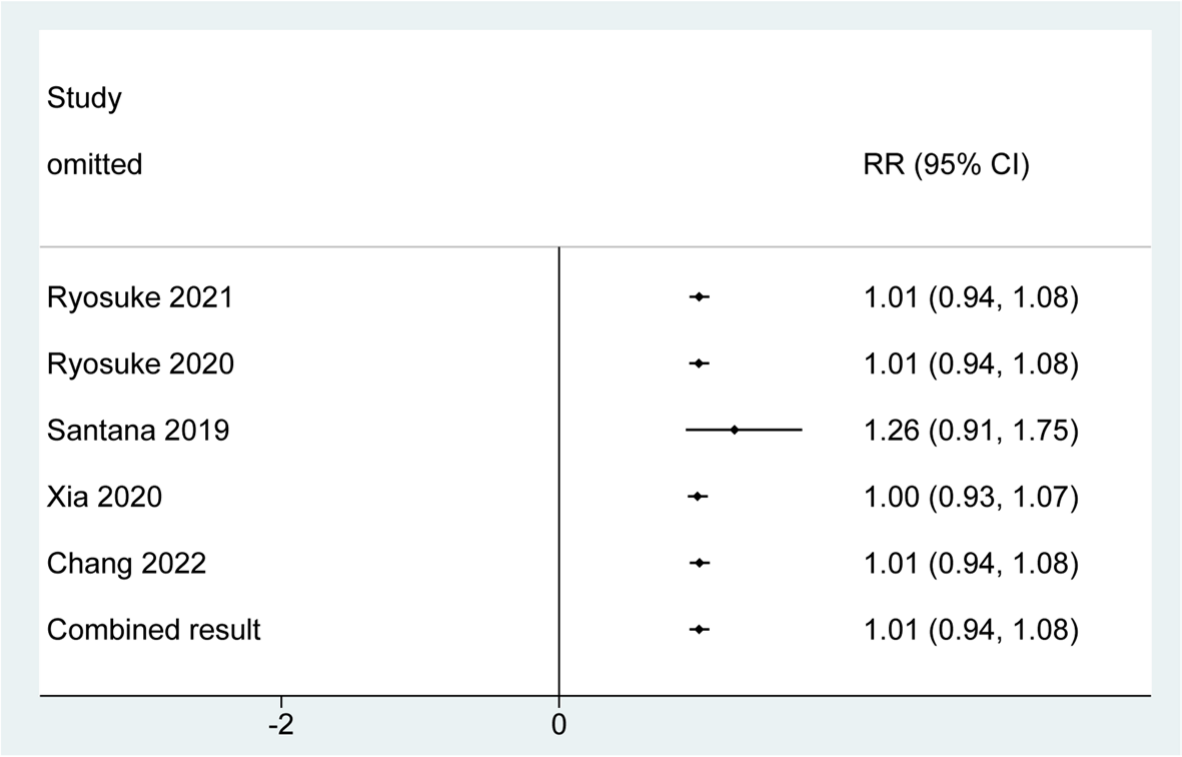
Sensitivity analysis based on the leave-one-out strategy showed that the exclusion of individual study did not significantly affect the result

A total of five studies with a total sample size of 783,626 were included. Heterogeneity assessment showed no significantly statistical heterogeneity among the five studies (*I*^*2*^ = 15.3%, *P* = 0.317), so we selected the fixed-effects model to perform meta-analysis. Pooled result from meta-analysis showed that there was no significantly statistical association between sarcopenia and the risk of myocardial infarction [RR = 1.01; 95% CI = 0.94, 1.08; *z* = 0.661, *P* = 0.509). The results of individual studies and pooled results are shown in Fig. 2. Furthermore, as shown in Fig. 3, the results of sensitivity analysis using the leave-one-out strategy showed that the pooled result of the current meta-analysis was robust.

### Publication bias

**Fig. 4:**
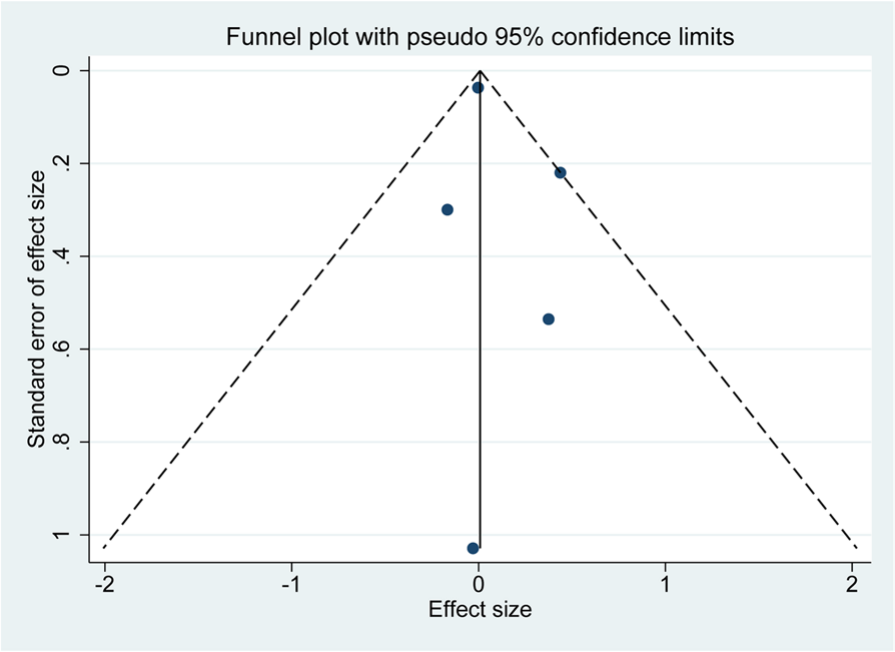
Funnel plot showed no publication bias for this meta-analysis

A funnel plot was evaluated for all five studies, and the results are shown in Fig. 4. The two sides of the funnel are symmetrical, and no significant publication bias was found in this study.

## Discussion

As far as we know, this is the first study to investigate the association between sarcopenia and myocardial infarction using the meta-analytical technique. In the current systematic review and meta-analysis, a total of 783,626 patients from 5 eligible studies were included in the final analysis. All five studies were high quality in methodological quality, while pooled result showed no significant association between sarcopenia and myocardial infarction.

Healthcare systems are under great stress with an increasing demand resulting from the ageing of the population [34], because various potential physiological changes and a higher risk of diseases emerge with ageing [35]. Sarcopenia and weakness are two common geriatric syndromes [2] and these two conditions are closely related [36]. Despite the fact that these two diseases were commonly confused, they are two distinct diseases [37]. It should be highlighted that sarcopenia and frailty are reported to be associated with chronic diseases [38] (e.g., chronic obstructive pulmonary disease [COPD] [39] and osteoporosis [40]), a high occurrence of polypharmacy [41, 42] and adverse events as falls and poor QoL [7, 8, 43].

Fortunately, sarcopenia and frailty are both curable, and their expenses can be avoided, eliminating pressing social needs in the future [44–47]. Clarifying how these two factors relate to chronic diseases is therefore essential [38]. Studies attempted to investigate the correlation between cardiovascular mortality and sarcopenia and frailty given that cardiovascular illnesses are a major worldwide disease causing disability and death [11–13], but the results have been inconsistent. Sarcopenia, however, is not significantly linked to the occurrence of myocardial infraction, according to the current systematic review and meta-analysis. In fact, among the five studies included in the current systematic review and meta-analysis, only one study [32] found that sarcopenia increased the risk of myocardial infraction, while the remaining four studies [28–31] did not reveal a significant association between sarcopenia and myocardial infraction. Age, the presence of a disease, dietary status, and lifestyle choices are all thought to have an impact on the development of sarcopenia. So far, sedentary and inactive lifestyles are known risk factors for the condition [2]. In these eligible studies included in the current systematic review and meta-analysis, patients with different diseases were involved, which will inevitably confuse the association between sarcopenia and myocardial infraction. Therefore, we also suggest to interpret our finding with caution before more studies with large-scale validate it.

This study has some limitations. There is a lack of research literature on the association between sarcopenia and myocardial infarction, which might contribute to the low confidence of the findings. In addition, it was not possible to perform subgroup analyses. The association of body mass and nutritional indicators with myocardial infarction in patients with sarcopenia should be explored in greater depth. Furthermore, we did not have strong confidence in whether publication bias influenced the pooled result as only 4 studies were included in the final analysis. Finally, there was no formal protocol for current systematic review and meta-analysis, thus inevitably compromising the transparency of the current study despite being conducted strictly in accordance with the PRISMA statement.

## Conclusions

Based on the limited evidence, we conclude that there is no significantly statistical association between sarcopenia and the occurrence of myocardial infarction. However, considering the extremely limited number of included studies, more in-depth studies are needed.

## Supplementary Information

**Additional file 1: Supplementary Table 1. **Details of search strategy of three target databases. **Supplementary Table 2. **The list of excluded studies during the full text screening phase.

## Data Availability

All data generated or analysed during this study are included in this article.

## Abbreviations

RR: Risk ratio
QoL: Quality of life
PRISMA: Preferred reporting items for systematic reviews and meta-analyses
CI: Confidence interval
AWGS: Asian working group for Sarcopenia
ASMI: Appendicular skeletal muscle mass index
ECG: Electrocardiogram
COPD: Chronic obstructive pulmonary disease
NOS: Newcastle Ottawa scale
