# Supplementary material for: A systematic review and meta-analysis of the association between sarcopenia and myocardial infarction
Source: BMC Geriatr. 2023 Jan 6;23:11. doi: 10.1186/s12877-022-03712-1 (PMC9825023; doi:10.1186/s12877-022-03712-1)
Supplement: Supplementary file 1 — Additional file 1: Supplementary Table 1. Details of search strategy of three target databases. Supplementary Table 2. The list of excluded studies during the full text screening phase. [file 12877_2022_3712_MOESM1_ESM.docx]

**Supplementary Table 1.** Details of search strategy of three target databases.

1. **PubMed**

| #No. | Search Details | Results |
| --- | --- | --- |
| #1 | "Myocardial Infarction"[MeSH Terms] | 190,385 |
| #2 | "myocardial infarction"[Title/Abstract] OR "myocardial infarctions"[Title/Abstract] OR "cardiovascular stroke"[Title/Abstract] OR (("cardiovascular system"[MeSH Terms] OR ("Cardiovascular"[All Fields] AND "system"[All Fields]) OR "cardiovascular system"[All Fields] OR "Cardiovascular"[All Fields] OR "cardiovasculars"[All Fields]) AND "Strokes"[Title/Abstract]) OR "myocardial infarct"[Title/Abstract] OR "myocardial infarcts"[Title/Abstract] OR "heart attack"[Title/Abstract] OR "heart attacks"[Title/Abstract] OR "acute coronary syndrome"[Title/Abstract] | 250,334 |
| #3 | #1 or #2 | 304,352 |
| #4 | "Sarcopenia"[MeSH Terms] | 8,258 |
| #5 | "Sarcopenia"[Title/Abstract] OR "Sarcopenias"[Title/Abstract] | 14,287 |
| #6 | #4 or #5 | 15,235 |
| #7 | #3 and #6 | 59 |

1. **EMBASE**

| #No. | Query | Results |
| --- | --- | --- |
| #1 | 'myocardial infarction':ti,ab,kw OR 'myocardial infarctions':ti,ab,kw OR 'cardiovascular stroke':ti,ab,kw OR 'cardiovascular strokes':ti,ab,kw OR 'myocardial infarct':ti,ab,kw OR 'myocardial infarcts':ti,ab,kw OR 'heart attack':ti,ab,kw OR 'heart attacks':ti,ab,kw OR 'acute coronary syndrome':ti,ab,kw | 368,546 |
| #2 | 'heart infarction'/exp | 447,800 |
| #3 | #1 OR #2 | 525,390 |
| #4 | sarcopenia:ti,ab,kw OR sarcopenias:ti,ab,kw | 21,653 |
| #5 | 'sarcopenia'/exp | 17,729 |
| #6 | #4 OR #5 | 24,134 |
| #7 | #3 AND #6 | 234 |
| #8 | #7 AND [embase]/lim AND 'human'/de | 218 |

1. **The Cochrane library**

| #No. | Search | Hits |
| --- | --- | --- |
| #1 | (Myocardial Infarction):ti,ab,kw OR (Myocardial Infarctions):ti,ab,kw OR (Cardiovascular Stroke):ti,ab,kw OR (Cardiovascular Strokes):ti,ab,kw OR (Acute coronary syndrome):ti,ab,kw | 43,490 |
| #2 | (Myocardial Infarct):ti,ab,kw OR (Myocardial Infarcts):ti,ab,kw OR (Heart Attack):ti,ab,kw OR (Heart Attacks):ti,ab,kw | 7,602 |
| #3 | #1 or #2 | 45,841 |
| #4 | MeSH descriptor: [Myocardial Infarction] explode all trees | 11,858 |
| #5 | #3 or #4 | 45,907 |
| #6 | (Sarcopenia):ti,ab,kw OR (Sarcopenias):ti,ab,kw | 1,915 |
| #7 | MeSH descriptor: [Sarcopenia] explode all trees | 644 |
| #8 | #6 or #7 | 1,915 |
| #9 | #5 and #8 | 17 |

**Supplementary Table 2.** The list of excluded studies during the full text screening phase.

| References | Reasons |
| --- | --- |
| 1. Kobayashi H, Takahashi M, Fukutomi M, Oba Y, Funayama H, Kario K: The long-term prognostic factors in hemodialysis patients with acute coronary syndrome: perspectives from sarcopenia and malnutrition. Heart and Vessels 2021, 36(9):1275-1282. 2. Nilsson G, Hedberg P, Leppert J, Ohrvik J: Basic anthropometric measures in acute myocardial infarction patients and individually sex- and age-matched controls from the general population. Journal of Obesity 2018, 2018. 3. Zhang N, Zhu W, Liu X, Chen W, Zhu M, Sun X, Wu W: Related factors of sarcopenia in hospitalized elderly patients with coronary heart disease. Chinese Journal of Cardiology 2019, 47(12):979-984. | Unrelated to topic |
| 1. Qiao YS, Tang X, Chai YH, Gong HJ, Zhang X, Stehouwer CDA, Zhou JB: Association of sarcopenia and a body shape index with overall and cause-specific mortality. Frontiers in Endocrinology 2022, 13. | Lack of grouping MI |

MI, myocardial infarction.
